# Atomic Force Microscopy Reveals Complexity Underlying General Secretory System Activity

**DOI:** 10.3390/ijms24010055

**Published:** 2022-12-20

**Authors:** Dylan R. Weaver, Gavin M. King

**Affiliations:** 1 Department of Physics and Astronomy, University of Missouri, Columbia, MO 65211, USA; 2 Department of Biochemistry, University of Missouri, Columbia, MO 65211, USA

**Keywords:** single molecule biophysics, AFM, membrane, protein, precursor, conformation

## Abstract

The translocation of specific polypeptide chains across membranes is an essential activity for all life forms. The main components of the general secretory (Sec) system of *E. coli* include integral membrane translocon SecYEG, peripheral ATPase SecA, and SecDF, an ancillary complex that enhances polypeptide secretion by coupling translocation to proton motive force. Atomic force microscopy (AFM), a single-molecule imaging technique, is well suited to unmask complex, asynchronous molecular activities of membrane-associated proteins including those comprising the Sec apparatus. Using AFM, the dynamic structure of membrane-external protein topography of Sec system components can be directly visualized with high spatial-temporal precision. This mini-review is focused on AFM imaging of the Sec system in near-native fluid conditions where activity can be maintained and biochemically verified. Angstrom-scale conformational changes of SecYEG are reported on 100 ms timescales in fluid lipid bilayers. The association of SecA with SecYEG, forming membrane-bound SecYEG/SecA translocases, is directly visualized. Recent work showing topographical aspects of the translocation process that vary with precursor species is also discussed. The data suggests that the Sec system does not employ a single translocation mechanism. We posit that differences in the spatial frequency distribution of hydrophobic content within precursor sequences may be a determining factor in mechanism selection. Precise AFM investigations of active translocases are poised to advance our currently vague understanding of the complicated macromolecular movements underlying protein export across membranes.

## 1. Introduction

The genetic code and associated molecular machinery produce several thousand distinct proteins in *Escherichia coli* [1]. In order to properly function, roughly one-third of these proteins need to enter into or pass across the cytoplasmic membrane. The general secretory (Sec) system provides the main export pathway. Proteins are transported across membranes as precursors, i.e., the mature domain with an N-terminal extension known as the signal sequence [2,3]. In the conventional view, the signal sequence targets the precursor to the membrane; recent work has shown that the mature regions are also likely involved [4]. The main translocation pathway through the membrane is provided by an integral membrane protein known as the translocon, SecYEG (73 kDa), which has homologs across all life (Sec61 in eukaryotes). The central cavity of translocon provides a channel that is wide enough to accommodate unfolded (i.e., without stable tertiary structure) precursor proteins as they transit through the membrane. A peripheral adenosine triphosphatase enzyme SecA (102 kDa) binds to SecYEG forming an ATP-driven translocase that is capable of deterministically translocating a diverse array of precursor proteins across the membrane. In addition, SecDF (81 kDa) is an ancillary subcomplex of the system that likely couples proton motive force into the translocation reaction, independent of hydrolysis performed by SecA [5].

A critical framework for the field is provided by a number of high-resolution structures of the main components of the system including SecYEG, SecA, and SecDF [6,7]. Despite this impressive knowledge base, the relative absence of dynamic structural information has left significant questions about the mechanism of precursor translocation unanswered. It is currently debated, for example, if the Sec system employs a power stroke mechanism or if a Brownian ratchet mechanism is at play [8,9,10]. 

Single-molecule methods are well suited to resolve asynchronous molecular activities such as those underlying protein translocation across membranes. Various single-molecule methods including optical trapping microscopy and smFRET are capable of dissecting mechanisms of macromolecular activity, and experiments on the Sec system have begun in earnest [9,10,11,12,13]. However, the number of proteins involved (at a minimum: SecYEG, SecA, precursor) as well as their variable stoichiometry presents complications [14,15,16]. For example, components can dissociate from translocase complexes during a measurement non-productively. The chemo-mechanical coupling of the system is not tight [17,18], likely due to non-productive hydrolysis. In the face of these challenges, high-precision atomic force microscopy (AFM) studies can provide a comprehensive view of dynamic multi-component protein complexes at work at membrane interfaces. Our laboratory and others have utilized AFM to analyze the Sec system, achieving molecular-scale (~10 Å) lateral resolution coupled with ~1 Å vertical resolution. In addition to two-dimensional imaging, kymographs (trace/retrace line scan analysis) have been used to achieve higher temporal resolution (<100 ms). 

### AFM Studies of Sec System in Near-Native Conditions

In this mini-review, we discuss recent AFM studies of core components of the general secretory system of *E. coli*, first in isolation, then co-assembled, and finally when translocating precursor proteins. The imaging modality of AFM is spotlighted here, specifically the tapping mode in fluid imaging [19]. The reader is directed elsewhere for a detailed discussion of sample preparation and analysis methods [20,21]. Likewise, force spectroscopy work on the Sec system via AFM or other tools can be found in other reports [22,23,24]. Unlike techniques such as crystallography or cryo-electron microscopy, biological AFM is carried out in an aqueous buffer solution at room temperature. The activity of biological macromolecules can be maintained under such conditions. In the case of the Sec system, we have verified this activity by adapting solution-based ATPase and translocation assays for samples prepared for AFM [15,17,25].

## 2. AFM Results

### 2.1. Imaging Isolated Components 

A logical first step when studying large multi-protein complexes, such as the general secretory system, is to image each component in isolation. This allows detailed topographic characterization of conformations and conformational dynamics, which can be used as a foundation for the interpretation of more topographically complex co-assembled systems. Here we discuss AFM imaging results for SecYEG, SecA, and SecDF when studied in the absence of protein binding partners.

#### 2.1.1. SecYEG

**Figure 1:**
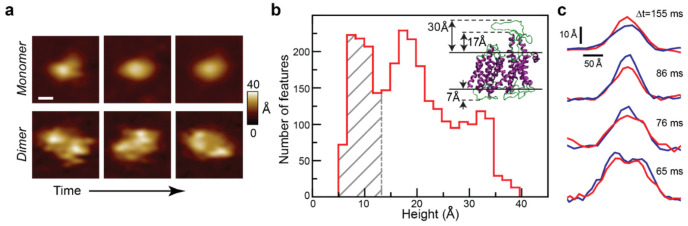
Membrane-external cytoplasmic protrusion dynamics of translocon SecYEG. (**a**) Successive AFM images of monomeric and dimeric features are displayed. The scale bar is 50 Å and applies to all images. The elapsed time between image frames was 180 s. (**b**) Histogram of the maximum height of individual SecYEG features (*N* = 2766, bin size = 1.7 Å). Grey hatched region represents periplasmic SecYEG with one major peak (~7 Å). In contrast, the cytoplasmic side of SecYEG exhibited two peaks (~17 Å and ~32 Å), with a substantial population of features with heights in between. (**b**, inset): crystal structure (PDB code: 3DIN) of SecYEG indicating asymmetry. The maximum height of the periplasmic protrusion is ~7 Å. In contrast, it is ~30 Å on the cytoplasmic side. The ends of the 8–9 helices extend ~17 Å above the lipid bilayer. Membrane boundaries are indicated (solid black lines). (**c**) Pairs of individual line scans are displayed (Red: trace; blue: retrace). The time difference between trace and retrace is shown for each pair of profiles. Data adapted from Ref. [16].

Our group was the first to apply AFM to image the core component of the general secretory system: translocon SecYEG. To mimic physiological conditions, purified SecYEG was reconstituted into liposomes extruded from *E. coli* polar lipid [26]. The resulting proteoliposomes were incubated on freshly cleaved mica to form supported lipid bilayers that are suitable for AFM imaging (Figure 1). These data, and others from our laboratory shown here, were collected on a commercial instrument (Asylum Research, Cypher). Care was taken to maintain a tip-sample force of <100 pN during imaging. We note that as an alternative to supported lipid bilayers, researchers have extended Sec system work to localized lipid patches known as nanodisks [27,28]. Distinct membrane-external conformations and dynamics can be directly observed and the oligomeric state of the reconstituted system evaluated. Reconstitution scrambles the orientation of SecYEG [16], so the first step in the analysis is the assignment of sidedness for individual SecYEG protrusions. The inherent asymmetry of SecYEG protrusions based on the crystallographic structure provides guidance for interpreting the experimentally measured distribution of maximum heights above the bilayer surface (Figure 1b). In particular, the loops protruding from the periplasmic side of SecYEG only extend 7 Å above the membrane surface, whereas the cytoplasmic loops are significantly taller (>17 Å). Thus, the first peak in the height histogram (Figure 1b, grey hatched region) can be assigned to the periplasmic side of SecYEG. The broad population of taller features (peaks at ~17 Å and ~32 Å as well as a shoulder between them) can be attributed to the cytoplasmic side of SecYEG undergoing conformational dynamics. These dynamics are likely due to extended and collapsed conformations of the unstructured loops that connect transmembrane (TM) helices 6–7 and 8–9. Sidedness assignment, based on the quantitative overlap between AFM measurements and crystallographic structures, has been corroborated by other means (e.g., see Section 2.2.1).

Representative AFM images of cytoplasmic SecYEG protrusions are shown (Figure 1a). The figure presents a time sequence of images of an individual monomer undergoing conformational dynamics in the top row, whereas the bottom row shows data for dimeric SecYEG. From frame to frame (Δt~180 s), the features exhibited significant changes in height, shape, and rotational orientation. Dynamics on a faster timescale (~100 ms) were measured by evaluating line scan data (Figure 1c). Here line scan pairs for four individual proteins are shown: trace (red, tip traveling left to right) and retrace (blue, tip traveling right to left). The measured height differences were 5, 8, 6, and 4 Å for each pair, respectively. Direct visualization of the disordered, flexible SecYEG structures in lipid bilayers provides a vista of the translocon in near-native conditions.

#### 2.1.2. SecA

As a peripheral membrane protein, SecA is found both in solution, crystallized in a number of distinct conformations [6,29], as well as in a membrane-bound state, characterized through a number of biophysical methods [22,30,31,32]. Our group imaged SecA in the presence of a lipid, which enhances its biochemical activity, and in the absence of a lipid [16,25]. Previous studies reported AFM images of SecA that had been dried in a desiccator [33,34]; such conditions are prone to artifacts. Our AFM experiments were carried out in fluid, allowing biochemical verification of ATPase activity [17,25].

**Figure 2:**
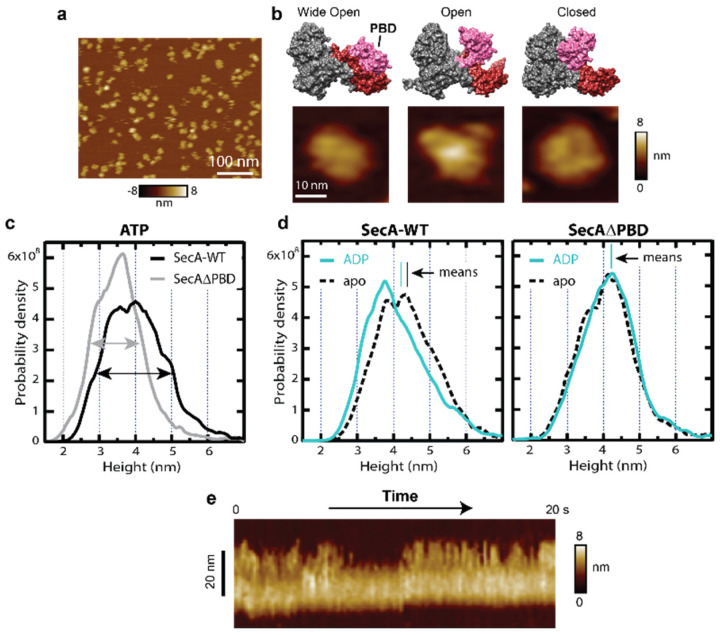
SecA dynamics and nucleotide-dependent topographical states. (a) AFM image of wild type SecA (SecA-WT) on mica in buffer conditions which favor monomer. (**b**) Structures of SecA in different conformational states are shown: the wide-open from *B. subtilis* (1M6N; left), open state from *B. subtilis* (1TF5; middle), and closed state from *T. maritima* (3DIN; right). The PBD and C-terminal segments are colored pink and red, respectively; all other domains are gray. Detailed AFM views of three SecA molecules exhibiting conformational differences are shown below the structures. (**c**) Height histograms for SecA-WT (*N* = 2437) and SecAΔPBD (*N* = 1458) in the presence of ATP. The FWHM (indicated with arrows) of the SecA-WT is 23 Å compared to 15 Å for the SecAΔPBD. (**d**) Height distributions for SecA-WT (apo: *N* = 3726; ADP: *N* = 6428) and SecAΔPBD (apo: *N* = 4009; ADP: *N* = 3104) in the presence and absence of ADP. Vertical lines show the means of the distributions. Only one mean is visible for SecAΔPBD because the apo and ADP means are identical to within uncertainty. (**e**) Kymograph of SecA-WT in saturating ATP over 20 s. The interval between adjacent line scans was Δ*t* = 95 ms. Data are adapted from Ref. [25].

Precursor transport through the SecYEG translocon is thought to be regulated by conformational changes of specific domains of SecA, but real-time, real-space measurement of these changes has eluded traditional structural methods. AFM has recently been used to visualize nucleotide-dependent conformations and conformational dynamics of SecA (Figure 2). Distinct topographical populations were observed in the presence of specific nucleotides [25]. The interpretation was aided by comparing wild-type SecA (SecA-WT) to a SecA mutant lacking the precursor-binding domain (SecAΔPBD). Statistically significant changes in the overall height and width of the mutant distributions in comparison to the wild type were apparent. The data in saturating ATP conditions revealed a 50% increase in full width at half maximum (FWHM) of SecA-WT compared to the mutant lacking the PBD domain (Figure 2c, compare grey and black arrows). This difference can be attributed to the PBD domain motion of SecA-WT. ADP binding shifted the height distribution for SecA-WT but did not alter the corresponding distribution for SecAΔPBD (Figure 2d). Hence, ADP binding induces a conformational change in PBD. By disabling the slow axis of the AFM tip raster scan, the time resolution can be enhanced significantly. The resulting kymograph analysis during basal ATP hydrolysis of SecA-WT revealed rapid, reversible transitions between a compact and an extended state at the ~100-ms time scale (Figure 2e). A conclusion from these studies is that ATP-driven SecA dynamics are largely due to PBD motion. 

#### 2.1.3. SecDF

**Figure 3:**
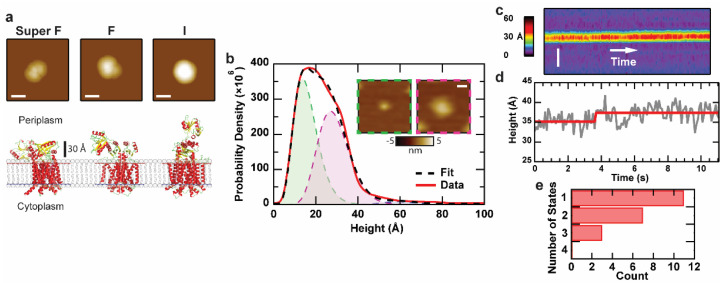
AFM imaging and kymograph analysis of SecDF in isolation. (**a**) Simulated AFM images of the periplasmic face of three SecDF structures: Super F, F, and I (5YHF, 3AQP, 5XAP respectively). The lateral scale bar is 10 nm, and pixel spacing is 5 Å. Bilayer background was determined based on the Orientations of Proteins in Membranes database. (**b**) Height distribution of SecDF protrusions (*N* = 15,957). The peaks for the three Gamma distributions used to fit the model are located at 14, 27, and 60 Å, respectively. (**b**, insets) Topographic asymmetry between the cytoplasmic (green dashed outline) and periplasmic (magenta dashed outline) face of SecDF. The lateral scale bar is 10 nm. (**c**) Representative kymograph and (**d**) corresponding height trace of an individual SecDF periplasmic domain, demonstrating minimal dynamics. The spatial axis is vertical (scale bar is 50 nm), and time is horizontal (90 ms per line), the grey line is the experimental data, the red line is the fit. (**e**) Histogram of the number of states detected across all SecDF kymographs (*N* = 21). Only protrusions with heights corresponding to the periplasmic domain are considered here. Data are adapted from Ref. [40].

SecDF, a heterodimeric integral membrane protein, is a factor in the Sec translocation process that stimulates the work of the translocase independent of ATPase SecA [35]. Along with translocon SecYEG and insertase YidC, SecDF is a central component of the holotranslocon complex [35,36]. Structural and functional analyses have indicated that the large periplasmic P1 domain of SecD plays a critical role in stimulating precursor protein transport in a manner dependent on proton motive force (PMF). High-resolution crystal structures demonstrate that SecDF can exhibit three distinct conformations, corresponding with P1 domain dynamics: the super membrane facing (Super F), membrane facing (F), and the intermediate (I) forms (Figure 3a) [37,38,39]. Despite the information that can be gathered based on these structures, there is still much speculation about the mechanism of action for SecDF and its interactions in the membrane.

Recently, we applied AFM imaging to study the sub-complex SecDF [40]. Similar to the analysis of the translocon, the topographic asymmetry of SecDF was directly visualized (Figure 3b). The data were fit using Bayesian information criterion (BIC) to determine the optimal number of models to utilize. The first two peaks (each 49% of the total) in the distribution occurred at approximately 14 Å and 27 Å, while the third and least populated peak (2% of the total) corresponded to a height of 60 Å. Comparison of these results with simulations based on crystal structure data allows the assignment of the 14 Å and 60 Å peaks to the cytoplasmic side and the periplasmic side in the I-state, respectively. The 27 Å peak could correspond to a compact conformation of the periplasmic face of SecDF similar to the Super-F state, an assertion that was verified via mutagenesis. 

**Figure 4:**
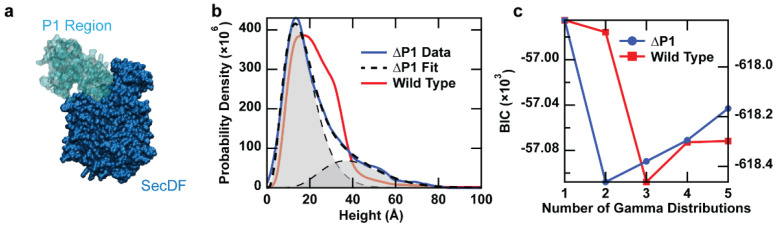
Mutagenesis of SecDF bolsters orientational assignment. (**a**) Structure of SecDF highlighting the P1 domain. The data is taken from a cryo-electron microscopy structure of the holotranslocon (5MG3). (**b**) Comparison of height distributions for the wild-type (red) and SecDFΔP1 mutant (*N* = 1490; blue). The overall shape of the distribution is altered significantly, and the number of distributions required to fit the data is decreased for the mutant. (**c**) BIC calculations for the optimal number of model distributions to fit the experimental data. Data are adapted from Ref. [40].

As an additional verification, proteoliposomes were prepared from a SecD mutant that lacked the P1 domain (SecDF∆P1). A direct comparison between SecDF-WT and SecDF∆P1 (Figure 4) shows that the peak at 27 Å, which made up for almost 50% of the wild-type population, was greatly reduced in the ΔP1 mutant. In addition, the number of distributions required to fit the mutant data was only two, instead of three. From this, one can have more confidence in assigning protrusion heights ≥27 Å as the periplasmic domain. A conceptual description of the comparison between simulations and mutations is described (see Section 2.2.2).

Analysis of the maximum height over time for SecDF protrusions (Figure 3c–e) shows minimal dynamics. Fitting revealed a single conformational state was the most prominent across all kymographs. A conclusion is that SecDF in isolation is in a highly compact and inactive/non-dynamic state. This motivates the co-assembly of SecDF with other components of the Sec system.

### 2.2. Imaging Co-Assembled Components

The activity of many Sec system components increases when the isolated macromolecules are allowed to interact with binding partners. For example, distinct ATPase activity levels have been characterized for SecA ranging from low: basal (isolated), to medium: translocase-activated (exposed to anionic lipid and SecYEG), to high: translocation-activated (exposed to anionic lipid, SecYEG, and precursor) [17,41]. Furthermore, the co-assembly of proteoliposomes containing SecYEG with SecA (SecYEG·SecA) increases the number of active translocons, raising the translocation activity of the reconstituted system to levels comparable to inner membrane vesicles which contain the full plethora of binding partners [16]. Recent work along these lines has also presented evidence that SecDF interacts with translocon SecYEG, altering membrane-external conformations and increasing conformational dynamics in a manner that is indicative of an activated system [40].

#### 2.2.1. SecYEG·SecA

**Figure 5:**
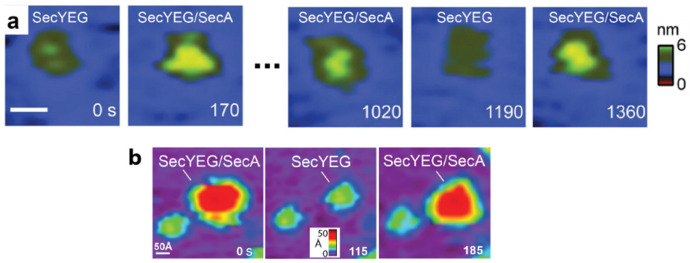
Direct visualization of the association and dissociation of translocase complexes SecYEG/SecA on two distinct supporting surfaces. (**a**) Image sequence on glass: at t = 0 s the cytoplasmic SecYEG protrusion is observed in the membrane. Indeed, 170s later, SecA binds, as indicated by the significant increase in protrusion height and volume. SecA dissociates at 1190 s. At 1360 s, SecA has associated with SecYEG. The scale bar is 100 Å. (**b**) Image sequence on mica: at 115 s, SecA dissociates from the membrane to reveal SecYEG beneath, and then SecA is seen to associate. Note that SecYEG in the bottom left remains unoccupied for all images, acting as a reference. The scale bar is 50 Å. Data are adapted from Refs. [15,43].

The complex comprising translocon SecYEG and SecA forms a translocase that is capable of driving precursors across the membrane in an ATP-dependent manner. This minimal system has been the subject of biochemical and biophysical investigations for decades [6]. Our group and others have provided direct visualization of SecYEG/SecA translocase association in a near-native membrane bilayer (Figure 5) [15,28,42,43]. Observations of SecYEG/SecA association and dissociation validate two important notions, further bolstering confidence in AFM results: (i) The events confirm SecYEG sidedness assignments. In binding to SecYEG, SecA effectively acts as a reversible and specific antibody identifying the cytoplasmic face of SecYEG. (ii) The binding events demonstrate that AFM imaging does not preclude critical cytoplasmic protrusion activities. The cytoplasmic SecYEG loops remain active for SecA binding despite AFM tip interactions inherent in AFM imaging. 

#### 2.2.2. SecYEG·SecDF

In the holotranslocon, SecDF comes into close proximity to the translocon SecYEG and is thought to position its periplasmic domain over the precursor exit site of SecYEG [35,36]. Notwithstanding SecDF-SecYEG proximity, studies have suggested that YidC mediates the binding between SecDF and SecYEG [5,44]. 

**Figure 6:**
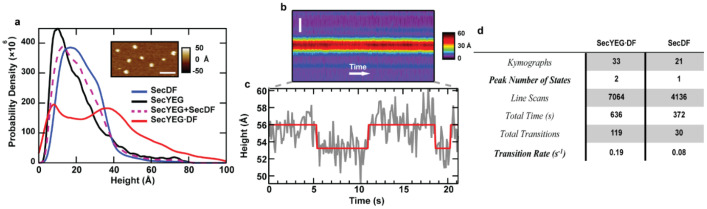
Basal conformational dynamics of SecDF co-assembled with SecYEG. (**a**) Smoothed histograms of membrane protein protrusions emanating from SecYEG alone (*N* = 3113; black), SecDF alone (*N* = 15,957; blue), the normalized summation of the previous two histograms (SecYEG + SecDF; purple dashed), or the co-assembled combination SecYEG∙DF (*N* = 10,118; red). (**a**, inset) AFM image showing several SecYEG∙DF features. The lateral scale bar is 100 nm. (**b**,**c**) Kymograph of a single SecYEG∙DF protrusion. The spatial axis is vertical (scale bar is 50 nm), and the time is horizontal (90 ms per line), the data is shown (grey) and the fit (red). (**d**) Summary of a number of states across all kymographs. SecYEG∙DF kymographs are more likely to exhibit two or more states, indicative of enhanced conformational dynamics in comparison to SecDF alone. Additionally, the co-assembled proteins displayed a greater number of transitions between individual states. Data are adapted from Ref. [40].

To gain insight into the potential interaction of SecDF with other Sec system components, we performed AFM experiments on proteoliposomes that contained co-assembled SecDF and SecYEG (SecYEG·SecDF) (Figure 6) [40]. We hypothesized that if SecYEG and SecDF do not interact, the histogram of SecYEG·SecDF membrane external protrusions will resemble a weighted sum of histograms for isolated proteins. What we noticed, in contrast, was a very broad distribution of heights for SecYEG∙DF (Figure 6a). In particular, the tall height shoulder that was very lightly populated beyond 40 Å with SecDF alone now has a significant population. In the presence of SecYEG, 9-fold more SecDF protrusions were found in an I-form-like conformation. These results are consistent with previous studies implicating chains in SecDF contacting SecY and SecG, giving rise to a functional state involved in precursor translocation across the membrane [45,46].

**Figure 7:**
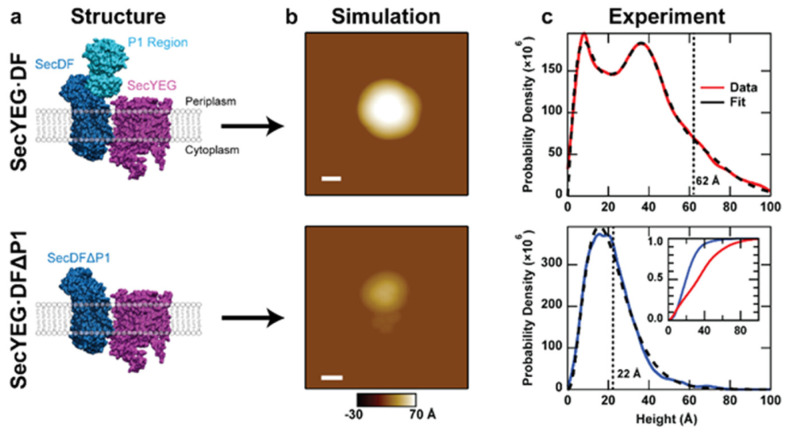
Analysis of co-assembled SecYEG·SecDF∆P1. (**a**) Models of wild-type and mutant SecDF co-assembled with SecYEG (structures manually oriented from 5MG3). Here, SecDF is shown in the intermediate (I) form in which the P1 domain is oriented upward. (**b**) Simulated AFM images of the periplasmic side of the structures are shown in (**a**). The lateral scale bar is 50 Å, and the pixel spacing is 4 Å. Note tip convolution obscures SecYEG from view in the case of the wild-type complex. (**c**) Height histogram and fit of AFM data consisting of individual SecYEG·SecDF molecules protruding from supported lipid bilayers. Both wild-type (*N* = 10,118) and ∆P1 mutant (*N* = 1088) samples were analyzed. Dashed vertical lines represent simulated maximum heights from (b). (**c**, inset) Integration of the wild type (red) and mutant (blue) histograms show the accumulated fraction. Data adapted from Ref. [40].

The broad nature of the SecYEG∙DF histogram is suggestive of conformational dynamics as transiently occupied conformational states cause broad shoulders in height distributions. Kymograph analysis of SecYEG∙DF protrusions confirmed this assertion (Figure 6b,d). The data show conformational states interconverting, with a >2-fold increase in the transition rate between states compared to SecDF alone (Figure 6d). This implies that SecDF is less prone to change conformations when subject to thermal driving forces if it is not allowed to associate with the translocon. The changing kinetics were commensurate with changes in the state number distributions. In the 10–20 s temporal windows provided by each kymograph, periplasmic SecYEG∙DF protrusions were most likely to be found in two distinct states (i.e., heights above the membrane surface). As a means to further demonstrate the dynamics of the P1 domain, we employed the mutant SecDF∆P1, and observed changes in the shape of the distribution, with no shoulder in the higher height range (Figure 7).

While this data supports the previous results indicating an interaction between SecYEG and SecDF, defining exactly how this interaction affects the mechanism of SecDF remains unclear. Modifications to the experiment, including introducing more members of the holotranslocon, are being considered as future directions for studying how SecDF fits into the current models for protein translocation, and the limitations of such studies are described below (see Section 3).

### 2.3. Imaging the Process of Precursor Translocation

With a knowledge base in place regarding the topographic signatures of individual and co-assembled Sec system components, we extended AFM studies to Sec system machinery undergoing active translocation. Two precursor species with different final destinations were employed. One is destined for the outer membrane: outer membrane protein A (pOmpA, alternatively known as proOmpA); and the other is destined for the periplasm, galactose-binding protein (pGBP).

#### 2.3.1. SecYEG·SecA with pOmpA or pGBP

Recent biochemical work has shown that aspects of translocation, including the apparent rate constant of the translocation reaction, depend on precursor species [18]. We postulated that differences in translocation reactions may also be apparent in the topography of active translocases. AFM imaging of SecYEG⋅SecA engaging pOmpA or pGBP was performed to evaluate this hypothesis.

- Defining two stages of the translocation reaction: initial and plateau

**Figure 8:**
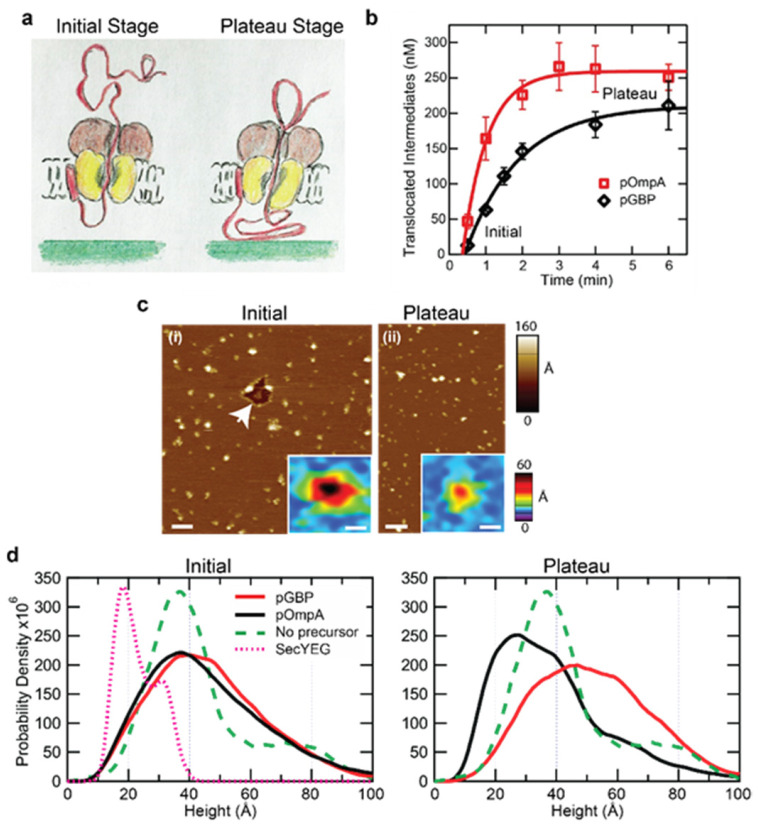
AFM study of Sec system at two stages of translocation. (**a**) Cartoon showing the main components: precursor (red) with an N-terminal signal sequence and a disulfide-stabilized loop, SecYEG (yellow), SecA (brown). Artwork credit: L.L. Randall. (**b**) Translocation activity of radiolabeled, oxidized pOmpA (red squares, n = 3) or oxidized pGBP (black circles, n = 3) assayed in vitro by protection from proteinase K. Error bars are SDs, and lines are fits to an exponential rise to maximum. (**c**) AFM images of Sec translocases with pOmpA at (i) the initial stage (30 s) of translocation, the arrow identifies a void in an otherwise continuous lipid bilayer, and (ii) at the plateau stage of activity (3 min). (**d**, left panel) Height distributions of active Sec translocases at 30 s with either pOmpA (red curve, number of features included, *N* = 12,387) or pGBP (black curve, *N* = 10,063). Data for translocases SecYEG·SecA with no precursor (green dashed curve, *N* = 587) as well as SecYEG alone (magenta dotted curve, *N* = 1875) are shown for reference. After reaching the plateau stage, the (**d**, right panel) height distributions of translocases engaged with pOmpA (red curve, *N* = 9565) or with pGBP (black curve, *N* = 6592) are shown. In all cases, translocation was halted by adding ADPAlF at the prescribed times. Note the vertical scale for SecYEG alone data was compressed two-fold in the height plots. Data are adapted from Ref. [15].

The starting material for all assays consisted of co-assembled SecYEG·SecA. To probe the translocase in the process of translocation, we used precursors containing disulfide-stabilized loops of either 44 or 59 aminoacyl residues near the C termini of pGBP and pOmpA, respectively. Translocation of oxidized precursors is initiated, but stalls at the loop, which is too large to pass through the channel in the translocon (Figure 8a). A time course of translocation for each precursor species is shown (Figure 8b). The time to reach the plateau was longer for pGBP (4.5 min) than it was for pOmpA (3 min).

To define the initial state of the system, translocation of either pOmpA or pGBP was allowed to proceed in saturating ATP for only 30 s, at which point ADP-AlF3 (ADPAlF) was added to halt further hydrolysis (Figure 8b, initial) [47]. To investigate translocase topography at the plateau stage, translocation proceeded for 3 min with pOmpA or 4.5 min for pGBP, at which point ADPAlF was added to halt further hydrolysis. For all reaction stages, immediately after ADPAlF addition, the reaction mix containing SecYEG·SecA, pOmpA or pGBP, and chaperone SecB was deposited onto freshly cleaved mica. We note that SecB, a 17 kDa soluble chaperone, was added to keep the precursor in a translocation-competent state [6]. Samples were incubated, rinsed, and then imaged in a physiological solution.

- Translocase topography varies with reaction stage

AFM imaging was carried out to follow the translocation reaction in a coarse-grained two-stage manner. One set of data was acquired at the initial stage of translocation and another set was acquired at the plateau stage. Representative AFM images of the Sec translocase engaging pOmpA at two stages are displayed (Figure 8c). The images contained many punctate protrusions corresponding to translocases and translocons above the *E. coli* lipid bilayer. Representative individual protrusions are shown as insets at the right corner of each image (Figure 8c). Topographic parameters were extracted and pooled together to construct kernel density estimates (smoothed histograms). Based on our previous work imaging SecYEG in lipid bilayers and SecYEG in complex with SecA in lipid bilayers [16,26,43], many of the protrusions exhibited topography commensurate with the translocase (i.e., a major population at about 37 Å [16]). In the absence of SecYEG, SecA does not appear to bind the lipid bilayer in a single preferred conformation [16], and often diffuses to the edges of bilayer patches which can act as sinks for diffusing species. Protrusions corresponding to the periplasmic side of SecYEG, which extend <10 Å above the membrane and do not engage SecA, were not included in the analysis [26]. Additionally, features exhibiting large heights (>100 Å), which were likely to be aggregates, were rare (<15% of the total) and not included in the analysis.

○ ***Similar topography at the initial stage***

Translocases engaging either pGBP or pOmpA exhibited very similar distributions of height above the membrane at the initial stage of the translocation reaction (Figure 8d, left panel, compare solid lines). The distributions for both precursors at the initial stage of translocation were maximally populated at the same height ~37 Å above the lipid bilayer. This population overlaps with the protrusion heights in proteoliposomes SecYEG·SecA in the absence of precursor (Figure 8d, green dashed curve). For translocases engaging precursors, the presence of a broad subpopulation with heights between 40 and 70 Å was common to both pGBP and pOmpA.

○ ***Different topography at the plateau stage***

We then investigated translocase topography at the plateau stage of translocation activity. In contrast to the largely precursor-independent initial stage, the height distributions of translocases at the plateau stage exhibited substantial differences (left and right panels of Figure 8d, right panel, compare solid lines). Many translocases engaging pOmpA displayed a large *decrease* in height at the plateau stage of translocation compared to the initial stage. The magnitude of height reduction is indicative of complete SecA dissociation, revealing just SecYEG. For pGBP, most translocases remained intact and exhibited an *increased* height at the plateau stage.

- Oligomeric state varies with ATP hydrolysis

**Figure 9:**
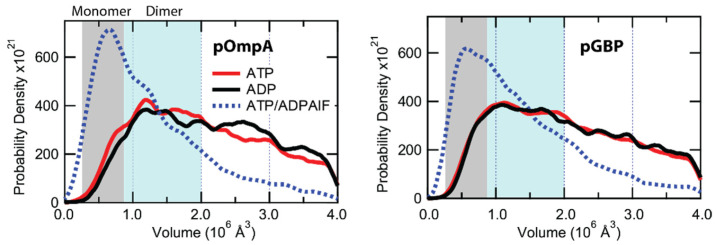
Oligomeric state changes with hydrolysis cycle. Volume distributions of translocases engaging pOmpA (left panel) with different nucleotides: ADP only (black curve, n = 1708), ATP only (red curve, n = 2518), or ATP followed by ADPAlF (blue dotted curve, n = 5088). Analogous volume distributions of translocases engaging pGBP (right panel) are also shown: ADP only (n = 2814), ATP only (n = 8441), or ATP followed by ADPAlF (n = 4973). The approximate monomer and dimer volume ranges of SecA are indicated by the gray-shaded region and blue-shaded region, respectively. Data are adapted from Ref. [15].

We also quantified the quaternary structure of SecA in the active translocases at the plateau stage of activity [15]. We analyzed translocases engaging either pOmpA or pGBP under different nucleotide conditions (Figure 9a). The volume distributions for ATP (red curve) or ADP (black curve) were broad with a peak around ~1 × 10^6^ Å^3^ for both precursors. The translocase volume distributions also exhibited a substantial population >2 × 10^6^ Å^3^. This population could consist of extended conformations of dimers or quaternary structures larger than dimers. Irrespective of this broad range, volumes were similar for translocases engaging both precursors with either ATP or ADP bound. On the basis of these data, we deduce that SecA often exists as a dimer during the hydrolysis state (ATP) and resting state (ADP), independent of the two precursor species tested. 

Volume analysis applied to translocases exposed to ATP followed by ADPAlF, which stabilizes the transition state, shows notably different results (Figure 9a, blue dotted curves). For both precursors, we observed a >2-fold increase in SecA monomer population for samples probed at the transition state (ADPAlF) compared to the hydrolysis state (ATP) or resting state (ADP). These observations indicate that the oligomeric state of SecA in the translocase changes during the ATP hydrolysis cycle.

- Conformational distinctions of dimeric translocases

**Figure 10:**
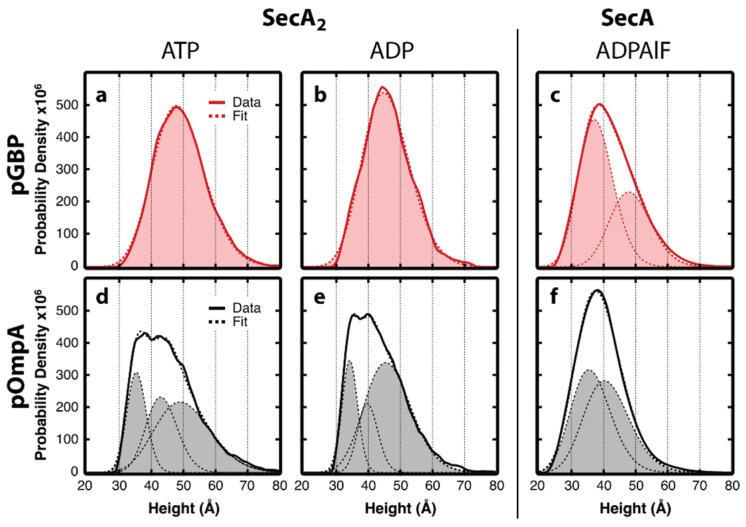
Translocase conformational changes with nucleotide. Height distributions for translocases engaging pGBP when exposed to (**a**) ATP (n = 3431), (**b**) ADP (n = 1128), or (**c**) ADPAlF (n = 1860), as well as translocases engaging pOmpA when exposed to (**d**) ATP (n = 1047), (**e**) ADP (n = 661), or (**f**) ADPAlF (n = 2112). Only translocases exhibiting volumes in the range corresponding to SecA_2_ or SecA monomer were included, as indicated. Bayesian information criterion was used to determine the optimal number of gamma distributions for each fit. The analysis prescribed one gamma distribution for (**a**,**b**), two for (**c**,**f**), and three for (**d**,**e**). Data are adapted from Ref. [15].

The volume analysis indicated that dimeric SecA occupied the translocon during the hydrolysis and resting states for both pOmpA and pGBP, but what about the conformation of these translocases? To provide insight, we focused on translocases in which SecA was in dimeric form. The resulting analyses with pGBP showed that in the presence of either ADP or ATP, translocases exhibited single-peaked height distributions (Figure 10a,b). In contrast, translocases engaging pOmpA exhibited multimodal height distributions under the same conditions (Figure 10d,e). To quantify these distinctions, we used Bayesian information criterion to determine the optimal number of model distributions to fit each dataset. This analysis prescribed one distribution for pGBP in either ATP or ADP. Three distributions were required for pOmpA subject to the same conditions. In addition to the different number of model distributions, the peak locations for pGBP were shifted higher (~5 Å) compared to pOmpA.

For both precursors, a primary difference between ATP and ADP appeared in an overall ~3-Å shift to higher heights in the presence of ATP, accompanied by an approximately 20% enhancement in the full width at half maximum of the height peaks. These changes are likely attributable to conformational dynamics associated with ATP hydrolysis. In particular, translocase conformations in the hydrolysis state and resting state varied substantially with precursor species on the time scale of AFM imaging. 

#### 2.3.2. Conclusions

**Figure 11:**
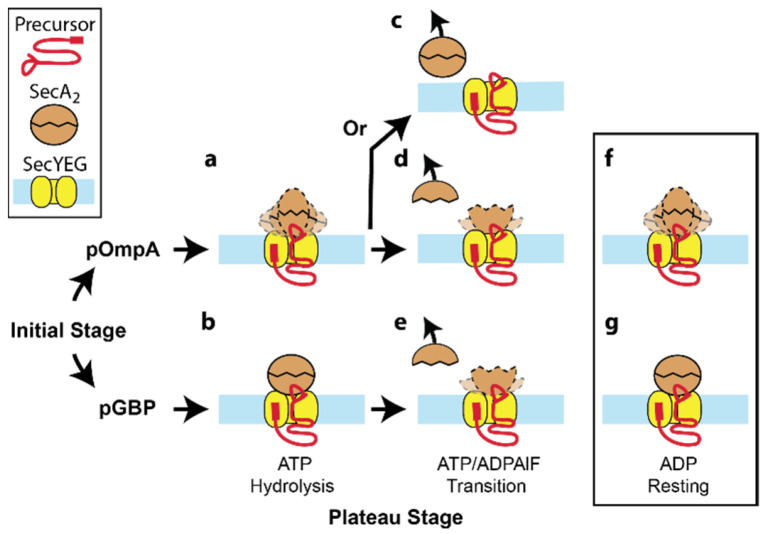
Model of precursor-dependent translocation. A summary of results is presented in cartoon form for pOmpA (**a**,**c**,**d**,**f**) or for pGBP (**b**,**e**,**g**) under the conditions listed. Individual components of the system include the following: precursor (red) with an N-terminal signal sequence and a disulfide-stabilized loop, SecA_2_ (brown), and SecYEG (yellow) in a lipid bilayer (light blue). The dashed lines indicate distinct stable conformations apparent on the time scale of AFM imaging. AFM measurements of the active Sec translocase do not provide direct visualization of SecYEG or the oligomeric state of SecYEG because it is buried underneath SecA. In the absence of this information, the monomer SecYEG is drawn in all conditions. In addition, cartoons are not meant to accurately convey the orientation of SecA or SecA_2_.

Three conclusions of our study of active Sec translocases are listed below and summarized graphically (Figure 11).

(i) SecA readily dissociates from the translocase when engaged with pOmpA, but the majority of SecA remains associated with the translocase when engaged with pGBP. It has been previously reported that SecA binds tightly to the SecYEG/pOmpA complex but is readily released upon ATP hydrolysis [14]. The results presented here are consistent with this notion. However, here, we show that the release of SecA is precursor-dependent.
(ii) The quaternary structure of SecA in the active translocase is dimeric (or higher order) in the hydrolysis (ATP) and resting (ADP) state and becomes monomeric in the transition state (ADPAlF). These changes in SecA quaternary structure were essentially independent of the two precursor species. Previous research has shown that dimeric SecA is prevalent [48,49], at least during the rate-limiting step of ATP hydrolysis, and that oligomeric state changes of SecA drive a critical step of the translocation process [50]. We showed that SecA_2_ in the translocase dissociates to monomer in the presence of the transition state analog ADPAlF, but remains dimeric (or higher order) in ATP or ADP. These results did not change substantially between the two precursor species that were studied. This is in agreement with previous work showing that precursor-stimulated ATP hydrolysis leads to SecA monomerization [7]. We note that the corresponding oligomeric states of SecYEG (or changes thereof) are largely concealed from the atomic force microscope tip by SecA in the AFM images.
(iii) Active translocases exhibit topographically distinct conformations that vary with precursor species on the time scale of AFM imaging. We attribute the conformational differences to SecA-driven structural rearrangements within the translocase.

AFM measurements provide structural corroboration for the significant precursor-dependent differences recently observed in the translocation reaction, including in the apparent rate constant and in the extent of precursor translocation [18]. Yet several questions remain unanswered. It will be interesting to identify the origin of the trimodal height distribution exhibited by translocases engaging pOmpA during hydrolysis (Figure 10d,e). SecA_2_ has been reported in at least three different orientations, and SecA protomers are thought to act with little cooperativity and slide or rotate about the interface [29,50]. Hence, the complex height distribution could be related to different orientational states of the SecA dimer interface which are employed during pOmpA translocation.

**Figure 12:**
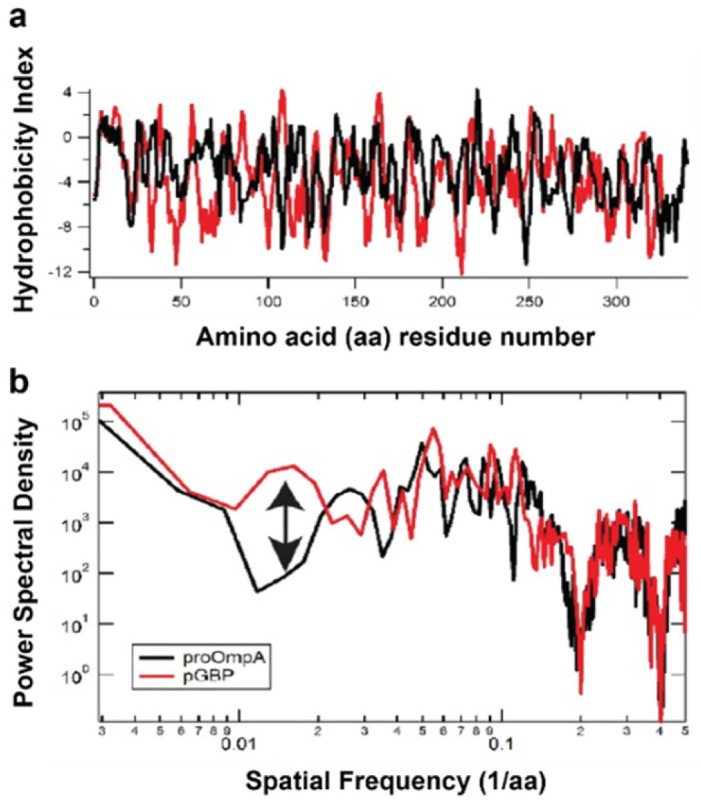
A distinction between pOmpA and pGBP in the periodicity of hydrophobicity. (**a**) Traditional hydrophobicity plots for pGBP (red) and pOmpA (black). (**b**) Fourier transform of hydrophobicity shows overall agreement between the two precursor species except around a spatial frequency of 0.017 1/aa (arrow).

What physical property of the precursor triggers the observed differences in translocase topography? The overall length and hydrophobicity of pOmpA and pGBP are similar (Figure 12a). One factor that could play a role is secondary structure content, which has been shown to modulate translocation efficiency [51], and differs between pGBP (37% helix 17% β-strand) and pOmpA (18% helix, 41% β-strand). Another is the periodic nature of the hydrophobic content. Taking the Fourier transform of the hydrophobicity curve provides a measure of the periodicity of hydrophobicity, showing how often hydrophobic content repeats per residue (amino acid, aa; proxy for linear distance) along the polypeptide chain. Fourier analysis reveals a significant difference between the two precursors around a spatial frequency of 0.017 1/aa (Figure 12b, arrow; note, scale is logarithmic). In particular, pGBP has an approximate 100-fold larger hydrophobicity signal than pOmpA around this frequency, which is well outside the expected distinctions in periodicity around 2 aa and 4 aa due to secondary structure differences [52]. It would be interesting to vary the spatial frequency of hydrophobic content of a model precursor sequence in a systematic manner to further evaluate this potential correlation with the translocation mechanism. 

A distinction between pOmpA and pGBP in the periodicity of hydrophobicity. (**a**) Traditional hydrophobicity plots for pGBP (red) and pOmpA (black). (**b**) Fourier transform of hydrophobicity shows overall agreement between the two precursor species except around a spatial frequency of 0.017 1/aa (arrow).

## 3. Limitations of These Studies

When evaluating experimental techniques, one must balance advantages *and* limitations. Here we discuss several shortcomings with respect to single molecule AFM studies of Sec system activities. 

Two common concerns affecting biological AFM are (i) the interaction force between the tip and the macromolecular sample and (ii) the interaction between the sample and the underlying supporting surface. The first concern can be alleviated via quantitative agreement analysis between AFM images and simulated images based on crystal structures [25]. Furthermore, observation of repeated association/dissociation of a peripheral component with its membrane-bound binding partner (e.g., SecA with SecYEG) can also alleviate this concern. For the Sec system, the second concern can be evaluated independently of AFM imaging by comparison between biochemical activity assays carried out in solution (i.e., with no surface nearby) with assays carried out in samples prepared for AFM. If observed, discrepancies can potentially be alleviated by altering the supporting surface. We found the chemo-mechanical coupling (ATP hydrolyzed per residue translocated) of the Sec translocase only varied twofold when comparing glass- supported samples to samples in solution; whereas the difference between mica and solution was more significant, likely due to increased confinement between the surface-proximal bilayer leaflet on the mica surface [17]. 

The topographic data of active Sec translocases presented here indicates that pGBP and pOmpA are transported across the cytoplasmic membrane by mechanisms that are distinct. However, there remains the formal possibility that all the mechanical steps required for translocation are the same for both precursors, but differing kinetics gives rise to our observations. During hydrolysis, the system could spend substantially more time in one conformation when engaging pGBP compared to pOmpA. Further work with higher time resolution will be required to distinguish between these possibilities. We also point out that our AFM experiments on stalled complexes at coarsely separated translocation stages were not designed to discriminate between translocation step models such as the power stroke mechanism and the Brownian ratchet. Future studies using continuous monitoring of individual translocases over time could provide insight into this fundamental question. 

AFM has been used to shed light on the interactions between translocon SecYEG and SecDF in supported lipid bilayers. A next step in this line of inquiry would be to image the full bacterial holotranslocon, incorporating SecYEG and SecDF along with SecA and insertase YidC. Yet the holotranslocon exhibits large membrane-external components on both sides of the bilayer: SecA on the cytoplasmic side and the P1 domain of SecD on the periplasmic side. Experience suggests that maintaining the full activity of the holotranslocon during AFM imaging will likely require a polymer cushion [53,54]. The challenge is to provide sufficient space for conformational dynamics and precursor motion without introducing undue instability or surface roughness which can limit spatial-temporal resolution. 

## 4. Outlook

Research carried out over the past decade has demonstrated that AFM imaging can uniquely characterize the activity of the general secretory system in near-native conditions. AFM provides access to conformational dynamics data in real time; this information complements traditional methods which are often limited to static snapshots. The comprehensive view of membrane activities that AFM makes available is well suited to unraveling complex pathways, such as the Sec system, which involves multiple proteins with time-varying stoichiometries undergoing difficult-to-synchronize dynamics. With recent advances in both AFM hardware and software, the method is poised to play an increasingly important role in unraveling fundamental Sec system mechanisms.
